# COVID-19 and coronary artery disease; A systematic review and meta-analysis

**DOI:** 10.1016/j.nmni.2023.101151

**Published:** 2023-05-23

**Authors:** Bahareh Hajikhani, Mahshid Safavi, Nazila Bostanshirin, Fatemeh Sameni, Mona Ghazi, Shahrooz Yazdani, Mohammad Javad Nasiri, Nafiseh Khosravi-Dehaghi, Negin Noorisepehr, Saba Sayyari, Masoud Dadashi

**Affiliations:** a Department of Microbiology, School of Medicine, Shahid Beheshti University of Medical Sciences, Tehran, Iran; b Department of Microbiology, School of Medicine, Alborz University of Medical Sciences, Karaj, Iran; c Department of Microbiology, Faculty of Medicine, Shahed University, Tehran, Iran; d Department of Cardiology, School of Medicine, Alborz University of Medical Sciences, Karaj, Iran; e Department of Pharmacognosy, School of Pharmacy, Alborz University of Medical Sciences, Karaj, Iran; f Evidence-Based Phytotherapy and Complementary Medicine Research Center, Alborz University of Medical Sciences, Karaj, Iran; g Department of Biotechnology, School of Medicine, Alborz University of Medical Sciences, Karaj, Iran; h Neonatal Health Research Center, Research Institute for Children's Health, Shahid Beheshti University of Medical Sciences, Tehran, Iran; i Non-Communicable Diseases Research Center, Alborz University of Medical Sciences, Karaj, Iran; j Shahid Beheshti University of Medical Sciences, Imam Hussein Hospital, Tehran, Iran

**Keywords:** COVID-19, Coronary artery disease, CAD, Meta-analysis

## Abstract

**Background and aim:**

Patients with underlying cardiovascular disorders such as coronary artery disease (CAD) are more prone to severe forms and multiple complications of COVID-19. The present systematic review and meta-analysis aimed to investigate the impact of CAD on patients with COVID-19.

**Methods:**

Main electronic databases, including Medline (via PubMed), EMBASE, and Web of Science, were carefully searched and reviewed for original research articles published between 2019 and 2021. One hundred nine studies that address CAD in patients with COVID-19 were selected and analyzed.

**Results:**

Following search and screening processes, 109 relevant publications were selected for analysis. The meta-analysis of prevalence studies indicated that the frequency of CAD among patients with COVID-19 was reported in 10 countries with an overall frequency of 12.4% [(95% CI) 11.1–13.8] among 20079 COVID-19 patients. According to case reports/case series studies, 50.9% of COVID-19 patients suffered from CAD. Fever was the most common symptom in these patients (47%); 36.5% also had hypertension.

**Conclusion:**

The results obtained during the present study show that the simultaneous presence of COVID-19 and CAD, especially in men and elderly patients, can increase the risks and complications of both diseases. Therefore, careful examination of the condition of this group of patients for timely diagnosis and treatment is strongly recommended.

## 1 Introduction

Coronavirus disease 2019 (COVID-19), caused by a coronavirus strain known as severe acute respiratory syndrome coronavirus 2 (SARS-CoV-2), has rapidly spread over the world, affecting billions of people [1,2]. Comorbidities affect a large percentage of COVID-19 patients [[3], [4], [5]]. Chinese studies revealed that 15–40% of patients with COVID-19 had a history of cardiac disease. Laboratory indicators of cardiac injury and cardiovascular involvement are found in 2–5 and 10–30% of patients, respectively [6,7]. A link between COVID-19 and cardiovascular disease (CVD) has been observed in clinical studies. COVID-19 patients with preexisting CVDs tend to have more severe complications and a higher mortality rate [8].

On the other hand, COVID-19 may also contribute to the development of CVD [7]. The high level of systemic inflammation linked to COVID-19 has been suggested to hasten the onset of subclinical problems or produce de novo cardiovascular damage, increasing the death risk in COVID-19 patients [9]. Different studies highlighted that the prevalence of cardiovascular conditions was higher in patients admitted to the intensive care unit (ICU) due to COVID-19 and those who died from this disease [6,10]. Coronary artery disease (CAD), the most common type of CVDs, is one of the major concerns of global health. As the third leading cause of mortality worldwide, it accounts for 17.8 million deaths annually [11,12]. Although CAD was thought to be caused by lipid accumulation, its pathophysiology is complex, and its exact underlying mechanisms are still unknown [13]. However, endothelial dysfunction may be the origin of this process, which is frequently caused by one or more of the following factors: stress, oxidative damage due to free radicals, genetic changes, persistent infection, or hypercholesterolemia [14]. Uncontrolled hypertension, diabetes, and smoking may also facilitate this process [15]. CAD symptoms range from asymptomatic, stable chest pain to acute coronary syndrome and sudden cardiac death [16]. Arrhythmias and heart failure are among the most common complications, and myocardial infarction (MI) is the most common manifestation of CAD. A coronary artery obstruction causes an inadequate blood supply to the heart, resulting in CAD symptoms [17]. Although patients with a history of cardiac disease appear to be more prone to become infected with SARS-CoV-2 and to have a more severe clinical course, their clinical features and outcomes have yet to be documented. Therefore, the present study aimed to investigate CAD in patients with COVID-19 through a comprehensive systematic review and meta-analysis.

## 2 Methods

### 2.1 Search strategy

A comprehensive systematic literature search was conducted by reviewing original research papers published in Medline, Web of Science, and Embase databases in May 2021. The following phrases were used in the search strategy: “COVID OR COVID-19 OR novel coronavirus OR new coronavirus OR coronavirus 2019 OR 2019-nCoV OR nCoV OR CoV-2 OR SARS-2 OR SARS-CoV-2 OR severe acute respiratory syndrome coronavirus 2”, AND “coronary artery disease OR CAD”. The search was restricted to original articles about CAD among patients with COVID-19. To identify further studies, bibliographies of related articles were also screened.

### 2.2 Inclusion and exclusion criteria

All case reports/case series and prevalence studies about CAD among patients with COVID-19 were evaluated. These studies reported sufficient data for analysis, such as the number of patients with COVID-19, the number of patients with CAD and SARS-CoV-2 infection, clinical symptoms, and laboratory findings. In the next step, two authors independently evaluated the recorded papers' titles, abstracts, and full texts based on the inclusion and exclusion criteria. The exclusion criteria were as follows [1]: studies considering CAD only [2], studies considering patients with COVID-19 only [3], review articles [4], abstracts presented in conferences, and [5] duplicate studies. After considering all studies based on inclusion and exclusion criteria, BH, MGH, and NKH selected appropriate papers.

### 2.3 Data extraction and definitions

In each study, the following items were considered: first author's last name, time of the study, time of publication, region, number of COVID-19 patients, number of COVID-19 patients with CAD, clinical symptoms, laboratory findings, outcomes, diagnostic methods, and treatment. The data were extracted by two independent individuals and verified by another researcher.

### 2.4 Meta-analysis

Statistical analysis was performed using STATA software, version 14.0 (Stata Corporation, College Station, Texas, USA) to report the frequency of CAD among patients with COVID-19. The fixed-effects (FEM) [18] and the random-effects models (REM) were utilized for data collection. Statistical heterogeneity was assessed using the Q-test and the I2 statistical methods. P-value <0.05 was considered statistically significant.

## 3 Results

### 3.1 Characteristics of included studies

**Fig. 1:**
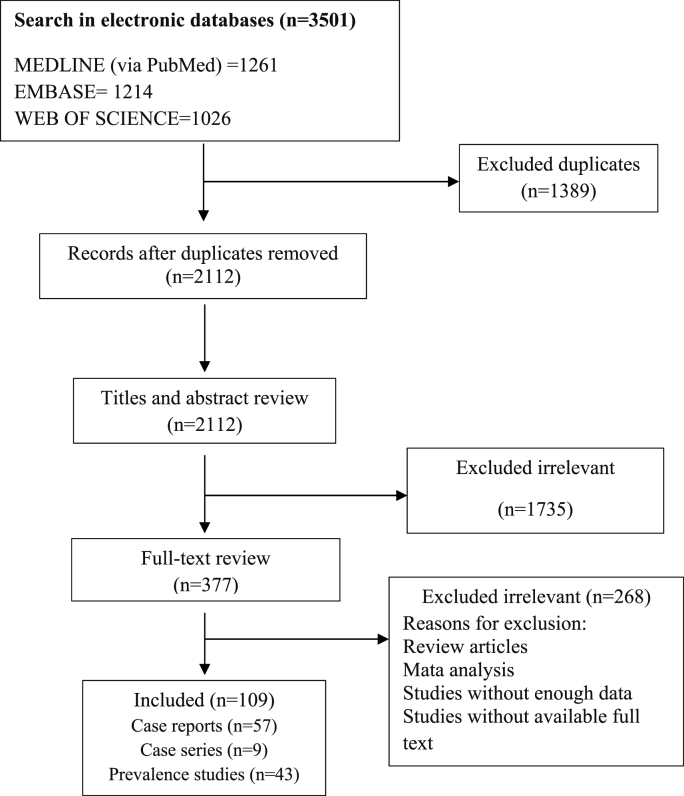
Flow chart of study selection for inclusion in the systematic review.

Overall, 3501 citations were recorded in the initial database searches. Three databases were searched, and therefore many duplicate studies were selected. After removing duplicates, 2112 non-duplicate studies remained. Of these, 1735 non-relevant studies were removed from our review after checking titles and abstracts. In the step of full-text screening, 268 irrelevant articles were also excluded. Eventually, 109 publications were selected for the final analysis (Fig. 1).

### 3.2 The frequency of CAD among patients with COVID-19 based on prevalence studies

**Fig. 2:**
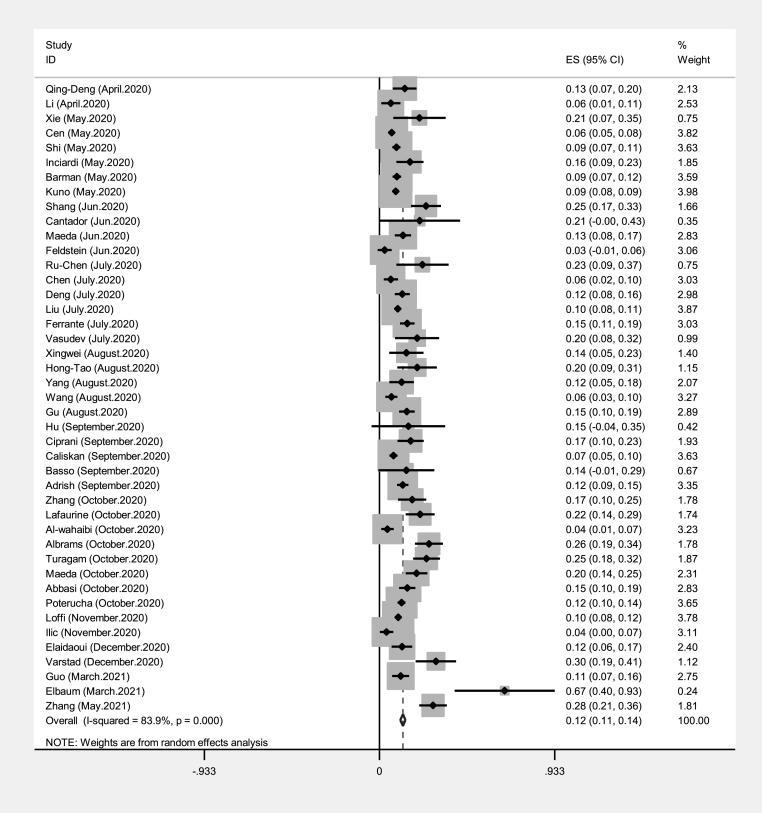
Forest plot of the meta-analysis on the prevalence of CAD among patients with COVID-19.

**Fig. 3:**
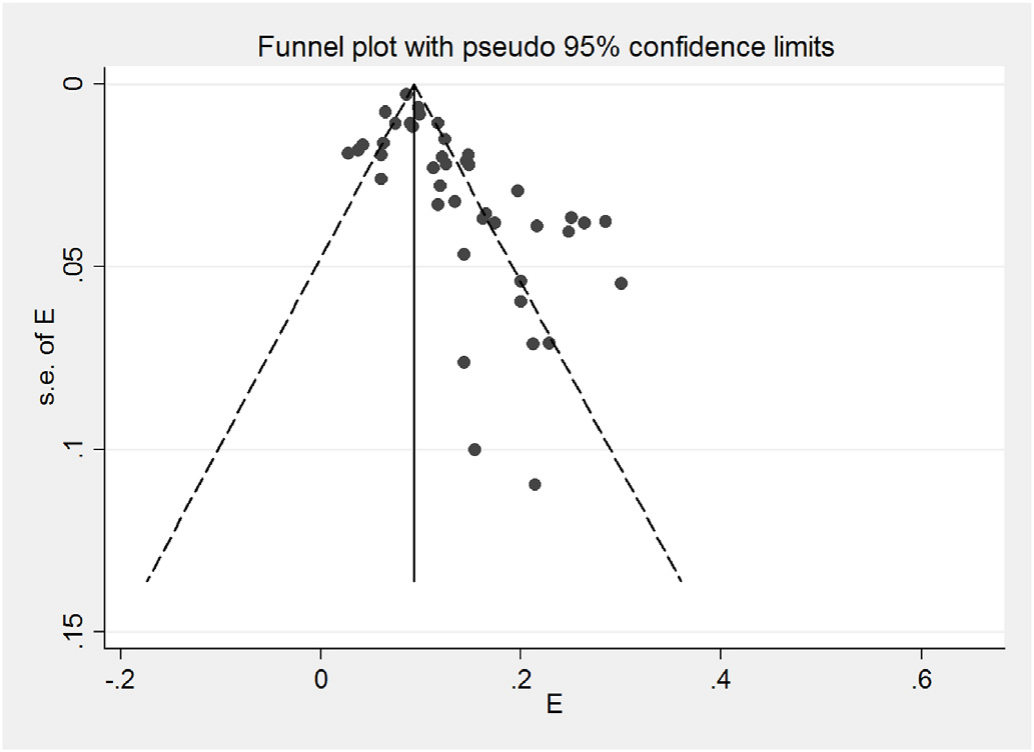
Funnel plot of the meta-analysis on the prevalence of CAD among patients with COVID-19.

**Fig. 4:**
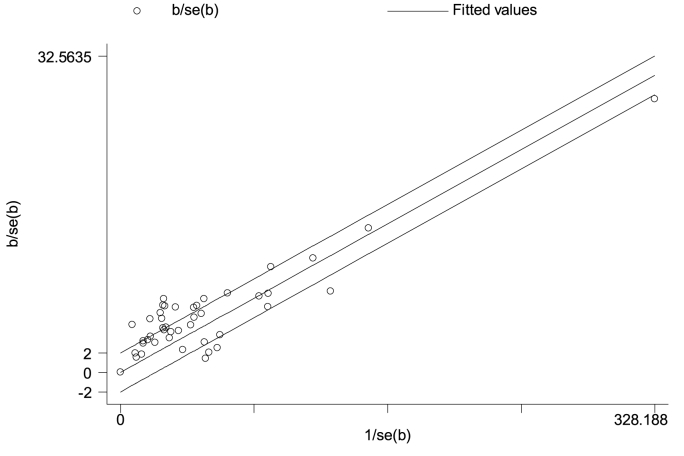
Galbraith of the meta-analysis on the prevalence of CAD among patients with COVID-19.

Out of 109 publications that reported CAD among patients with COVID-19, 43 articles (20 from Asia, 12 from America, and 11 from Europe) were prevalence studies, and 66 (12 from Asia, 26 from Europe, 27 from America, and one from Oceania) were case reports/case series (Table 1, Table 2). A meta-analysis of prevalence studies indicated that the frequency of CAD among patients with COVID-19 was reported in 10 countries (China, Oman, Italy, Norway, Turkey, Spain, Serbia, Morocco, France, and the USA) with an overall frequency of 12.4% (95% CI 11.1–13.8) among 20079 COVID-19 patients (Table 3). Fig. 2 summarizes almost all of the necessary data for a meta-analysis. The heterogeneity among assessed articles can be seen in Fig. 3, Fig. 4.

**Table 1:** Characteristics of included prevalence studies.

First author	Country	Published time	Number of patients with COVID-19	Number of COVID-19 patients with CAD	Mean age	Male/Female	Diagnostic method
Feldstein [39]	USA	Jun.2020	73	2	9	NR	RT-PCR
Kuno [40]	USA	May.2020	8438	723	58	NR	PCR
Elbaum [41]	USA	March.2021	12	8	60	M	RT-PCR
Vasudev [42]	USA	July.2020	45	9	61	NR	RT-PCR/Echocardiography
Turagam [43]	USA	October.2020	140	35	61	NR	RT-PCR
Maeda [44]	USA	July.2020	224	45	63	NR	PCR/Chest X-ray
Poterucha [45]	USA	October.2020	887	104	64	F(374)/M(513)	RT-PCR/electrocardiography
Maeda [46]	USA	October.2020	181	36	66	NR	RT-PCR
Abbasi [47]	USA	October.2020	257	38	67	NR	RT-PCR
Basso [48]	USA	September.2020	21	3	69	NR	PCR
Albrams [49]	USA	October.2020	133	35	N.R	NR	RT-PCR
Adrish [50]	USA	September.2020	469	58	NR	NR	RT-PCR
Li [51]	China	April.2020	83	5	43	NR	PCR
Al-wahaibi [52]	Oman	October.2020	143	6	49	M(124)/F(19)	RT-PCR
Guo [7]	China	March.2021	187	21		NR	RT-PCR
Cen [53]	China	May.2020	1007	65	61	NR	RT-PCR
Zhang [54]	China	May.2021	144	41	61	NR	RT-PCR
Chen [55]	China	July.2020	150	9	62	NR	PCR
Shi [56]	China	May.2020	671	60	63	NR	RT-PCR
Hong-Tao [57]	China	August.2020	55	11	63	NR	PCR
Wang [58]	China	August.2020	222	14	63	NR	RT-PCR
Zhang [59]	China	October.2020	98	17	63	NR	RT-PCR
Deng [60]	China	July.2020	264	32	64	NR	RT-PCR
Hu [61]	China	September.2020	13	2	64	M	RT-PCR
Qing-Deng [62]	China	April.2020	112	15	65	NR	RT-PCR/Echocardiography
Yang [63]	China	August.2020	94	11	66	NR	PCR
Gu [64]	China	August.2020	275	40	66	NR	PCR
Shang [65]	China	Jun.2020	113	28	67	NR	RT-PCR
Xingwei [66]	China	August.2020	56	8	68	NR	RT-PCR
Ru-Chen [67]	China	July.2020	35	8	69	NR	RT-PCR
Xie [68]	China	May.2020	33	7	NR	NR	RT-PCR/Chest tomography
Liu [69]	China	July.2020	2044	199	NR	NR	RT-PCR
Ilic [70]	Serbia	November.2020	107	4	39	NR	PCR/CT-scan
Caliskan [71]	Turkey	September.2020	565	42	48	NR	PCR/Tomography
Elaidaoui [72]	Morocco	December.2020	134	16	53	F(61)/M(73)	RT-PCR
Varstad [73]	Norway	December.2020	70	21	59	NR	RT-PCR
Inciardi [74]	Italy	May.2020	99	16	67	NR	PCR
Barman [75]	Turkey	May.2020	607	56	68	NR	RT-PCR
Cantador [76]	Spain	Jun.2020	14	3	73	NR	PCR
Lafaurine [77]	France	October.2020	111	24	74	M	RT-PCR/CT-scan
Loffi [78]	Italy	November.2020	1252	124	75	F(22)/M [102]	RT-PCR
Ciprani [79]	Italy	September.2020	109	18	N.R	NR	RT-PCR
Ferrante [80]	Italy	July.2020	332	49	NR	NR	RT-PCR

RT-PCR; Real time-Polymerase Chain Reaction.

NR; not report.

**Table 2:** Characteristics of case reports/case series studies.

First author	Country	Published time	Type of study	Number of patients with COVID-19	Number of COVID-19 patients with CAD	Mean age	Gender	Diagnostic method
Alabdulgader [81]	Saudi Arabia	Jan-2020	Case report	1	1	35	M	PCR/chest X-ray/Echocardiography
Sheikh [82]	USA	Dec-2020	Case report	1	1	56	M	Real-time PCR/electrocardiogram
Albiero [83]	Italy	May-2020	Case report	1	1	70	M	PCR
Ekinci [84]	Turkey	Dec-2020	Case report	1	1	57	M	PCR
Ali [85]	Australia	Dec-2020	Case report	1	1	59	M	PCR
Rapkiewicz [86]	USA	Jun-2020	Case series	7	7	57.4	3 ​M/4F	PCR
Azanza [87]	Mexico	Nov-2020	Case report	1	1	60	M	PCR
Lapergue [88]	France	Aug-2020	Case series	3	3	51.6	2 ​M/1F	PCR
shoman [89]	Qatar	Sep-2020	Case series	3	3	NR	NR	RT-PCR
Juthani [90]	USA	Jun-2020	Case report	1	1	29	M	PCR
Fisher [91]	USA	May-2020	Case report	1	1	81	F	RT-PCR/CT scan
Nelson [92]	USA	Sep-2020	Case report	1	1	15	F	PCR
Coyle [93]	USA	Jul-2020	Case report	1	1	57	M	PCR
Cogan [94]	Belgium	Jul-2020	Case report	1	1	19	M	PCR/echocardiography/tomography coronary angiography (CTCA)
Rothstein [95]	USA	Apr-2020	Case report	1	1	79	F	PCR/angiography
Celik [96]	Turkey	Jul-2020	Case report	1	1	45	M	RT-PCR/chest X-ray
Rozenshteyn [97]	USA	Oct-2020	Case series	2	2	57	2F	PCR/CT angiogram
Rivero [98]	Spain	May-2020	Case report	1	1	66	M	PCR/Echocardiography/Coronary angiogram
Fischer [99]	France	May-2020	Case report	1	1	63	M	PCR
Setia [100]	USA	Jun-2020	Case report	1	1	59	M	RT-PCR/CT Scan/Echocardiography/coronary angiography
Masood [101]	Emirates	Jun-2020	Case report	1	1	34	M	PCR/Chest radiography
Harari [102]	USA	May-2020	Case report	1	1	40	F	PCR
Kurdi [103]	Caucasus	Jan-2020	Case report	1	1	54	M	PCR/chest X-ray/Coronary angiogram/CT
Suryawan [104]	Indonesia	Sep-2020	Case report	1	1	85	M	RT-PCR/Echocardiography
Park [105]	Korea	Oct-2020	Case series	2	2	75.5	2F	PCR/chest X-ray/Echocardiography
Khalid [106]	USA	Aug-2020	Case report	1	1	48	M	RT-PCR
Kim [107]	Korea	Jul-2020	Case report	1	1	60	M	RT-PCR
Kireev [108]	Russian	Nov-2020	Case report	1	1	35	M	PCR/Echocardiography/CT scan
Kumar [109]	USA	Apr-2020	Case report	1	1	48	F	PCR
Leon [110]	Spain	Jun-2020	Case report	1	1	74	M	PCR
Tabaza [111]	USA	May-2020	Case report	1	1	59	F	PCR/Scan tomography/Echocardiography
Pelle [112]	Italy	Dec-2020	Case report	1	1	86	F	Real-time-PCR/CT Scan
Fabi [113]	Italy	Dec-2020	Case series	3	3	87	3 ​M	PCR/Coronary angiogram/Echocardiography
Marwa Soltani [114]	Canada	Jun-2020	Case report	1	1	63	F	PCR/Coronary angiogram/Echocardiography
Pasqualetto [115]	Italy	2020	Case series	3	3	83.3	2 ​M/1F	PCR/coronary angiography/CT Scan/Echocardiography
Ali [116]	USA	Sep-2020	Case report	1	1	27	M	PCR/Echocardiography/chest X-ray
Nakao [117]	Japan	Sep-2020	Case report	1	1	84	M	RT-PCR
Ong [118]	USA	Jul-2020	Case report	1	1	29	M	RT-PCR
Orr [119]	USA	2020	Case report	1	1	12	M	PCR, serology antibodies
Alexandra [120]	Switzerland	Nov-2020	Case report	1	1	22	M	PCR/ELISA/CT scan
Erquicia [121]	Spain	May-2020	Case report	1	1	64	M	RT-PCR/coronary angiography
Antuna [122]	Spain	Jul-2020	Case report	1	1	81	M	PCR
Lemos [123]	Brazil	Aug-2020	Case report	1	1	60	M	PCR
Ranard [124]	USA	Oct-2020	Case report	1	1	35	M	PCR, serology antibodies
Craver [125]	USA	May-2020	Case report	1	1	17	M	PCR
Uddin [126]	Croatia	2020	Case report	1	1	63	M	PCR
Rey [127]	Spain	Jun-2020	Case report	1	1	59	M	PCR
Salna [128]	USA	2020	Case report	1	1	57	M	RT-PCR
Farrokhran [129]	USA	2020	Case series	2	2	76	2F	RT-PCR/Echocardiography
Shams [130]	Qatar	2020	Case report	1	1	28	M	RT-PCR
Sharma [131]	UK	Jul-2020	Case report	1	1	50	M	PCR
Siddamreddy [132]	USA	Apr-2020	Case report	1	1	61	F	PCR
Singhavi [133]	India	Sep-2020	Case report	1	1	20	M	RT-PCR
Yandrapalli [134]	USA	Jul-2020	Case report	1	1	67	F	PCR/Chest radiology
Raut [135]	India	Aug-2020	Case report	1	1	5	M	PCR/Echocardiography
Jain [136]	USA	Sep-2020	Case series	2	2	10.5	2 ​M	RT-PCR/Chest radiography/Echocardiography
Nakamoto [137]	Japan	Aug-2020	Case report	1	1	50	M	PCR/Echocardiography/Chest X-ray
Proenca [138]	Portugal	Jan-2020	Case report	1	1	62	F	PCR/Echocardiography
Tavares [139]	USA	Oct-2020	Case report	1	1	38	M	PCR
Tedeschi [140]	Italy	Aug-2020	Case report	1	1	60	M	PCR
Thaibaud [141] Lacour	France	Apr-2020	Case report	1	1	68	M	PCR
Xuan [142]	China	May-2020	Case report	1	1	70	M	PCR/chest X-ray/Echocardiography
Villa [143]	Italy	2020	Case report	1	1	70	M	PCR
Wacker [144]	Switzerland	2020	Case report	1	1	10	M	PCR, serology antibodies
Yolcu [145]	Turkey	Apr-2020	Case report	1	1	55	M	PCR
Zendjebil(146)	France	Jul-2020	Case report	1	1	42	M	RT-PCR

RT-PCR; Real time-Polymerase Chain Reaction.

NR; not report.

**Table 3:** Frequency of CAD among patients with COVID-19.

COVID-19 patients with CAD			Prevalence% (95% CI)	Number of studies	Number of patients	I-squared
**Overall**			12.4 (11.1–13.8)	43	2068/20079	83.9%
**Subgroup**	***Asia***	China	11.7 (9.6–13.8)	20	373/3400	80.7%
		Oman				
	***Europe***	Italy	12.4 (9.6–15.2)	11	599/5799	81.2%
		Norway				
		Turkey				
		Spain				
		Serbia				
		Morocco				
		France				
	***America***	USA	10.8 (8.2–13.3)	12	1096/10880	81.2%

### 3.3 The frequency of CAD among patients with COVID-19 among different continents based on prevalence studies

The meta-analysis of prevalence studies showed that the frequency of CAD among patients with COVID-19 was 10.8% (95% CI 8.2–13.3) among 10880 patients in America, 12.4% (95% CI 9.6–15.2) among 5799 patients in Europe, and 11.7% (95% CI 9.6–13.8) among 3400 patients in Asia (Table 3). There was no data on CAD infection prevalence among COVID-19 patients from Africa and Oceania. As shown in Table 1, the most COVID-19 patients with CAD were reported in the USA.

### 3.4 The frequency of CAD among patients with COVID-19 based on case reports/case series

We assessed the CAD infection among cases with COVID-19, which was reported in the mentioned electronic databases. Characteristics of case reports/case series studies (which should have been considered during the analyses mentioned above) are shown in Table 2. According to these studies results, 84 (54.9%) CAD patients have been reported among patients with COVID-19 from 26 countries (Table 2). Most of the cases were in the USA (33 cases), Italy (10 cases), France (6 cases), and Spain (5 cases) (Table 2). Among the patients whose gender was mentioned, 24 patients with CAD were women, 58 were men, and one was unknown. Evaluation of case reports/case series revealed that out of 84 patients with COVID-19, 63 patients had the underlying disease; most were from America and Europe, respectively. The continents of Asia and Oceania were also in the next rank. It should be noted that at the time of this study, there were no reports of underlying disease in COVID-19 patients with CAD in Africa. According to the results of the present study, the most common underlying diseases were hypertension (31/84), diabetes (29/84), obesity (11/84), multisystem inflammatory syndrome (10/84), and dyslipidemia (10/84) (Table 4). In COVID-19 patients with CAD, the clinical symptoms were also considered. Thirty forms of clinical symptoms have been identified in these patients, depending on the findings of the investigations (Table 5). Of these, Fever (40/84), Cough (29/84), Chest pain (19/84), and dyspnea (21/84) were the most common clinical symptoms among COVID-19 patients with CAD. Of the 22 types of CAD registered in the studies, Acute coronary syndrome (8/84), Coronary Atherosclerosis, Acute thrombotic occlusion of the right coronary artery, stenosis of the proximal left anterior descending coronary artery (6/84 cases for each), and ST-elevation myocardial infarction (STEMI), Occluded artery (5/84 cases for each) were the most common types of CAD in CAD-COVID-19 patients (Table 6). Laboratory examination of patients showed that increased D-dimer (20.2%), Increased troponin (15.4%), elevated C-reactive protein (CRP) (14.2%), elevated ferritin (11.9%), elevated ESR (5.9%), high LDH, and elevated creatine kinase (each 4.7%), were the most common findings (Table 7). The study also looked at the patients' outcomes. According to the analyzed studies, 36 out of 84 patients returned to life, 22 patients died, and the outcomes of 26 patients were not reported. The drugs used in the case reports/case series articles for treating COVID-19 patients with CAD are summarized in Table 8. Umifenovir (5.9%), Remdesivir, Oseltamivir, Lopinavir, and Ritonavir (each 2.3%) were the most widely used antiviral drugs for the treatment of these patients. Among the antibacterial agents, Azithromycin (11.9%), Ceftriaxone (5.9%), and Piperacillin-Tazobactam (2.3%) were the most widely used drugs reported in 10, 5, and 2 studies, respectively. Several treatment regimens, including Hydroxychloroquine (HCQ) and azithromycin, have been used in some studies. Other used drug combinations are presented in Table 8 as “others”. As it turns out, Asprin (in 20 studies), HCQ (in 14 studies), clopidogrel (in 9 studies), and Enoxaparin, Tocilizumab (6 studies for each) were other drug combinations that were used more than others in the treatment of patients.

**Table 4:** Underlying conditions of patients evaluated in included studies.

Variable	No of study	n/N	%
Acute coronary syndrome	2	2/84	2.3
Asthma	2	2/84	2.3
Smoking	5	5/84	5.9
Bladder neoplasm	1	1/84	1.2
Conjunctivitis	1	1/84	1.2
Lung cancer	1	1/84	1.2
Prostate cancer	1	1/84	1.2
Renal cell carcinoma	1	1/84	1.2
Hypertension	23	31/84	36.5
Type 2 diabetes	23	29/84	34.1
Obesity	7	11/84	12.9
Multisystem inflammatory syndrome	7	10/84	11.7
ARDS^a^	1	1/84	1.2
Coronary artery dilatation	1	1/84	1.2
Myocardial infarction	1	1/84	1.2
Dyslipidemia	10	10/84	11.7
Normocytic hypochromic anemia	1	1/84	1.2
Ischemic stroke	4	4/84	4.7
Stress cardiomyopathy	2	2/84	2.3
Myocarditis	1	1/84	1.2
Myocardial infarction	1	1/84	1.2
Ventricular dysfunction	1	1/84	1.2
Cardiomyopathy	1	1/84	1.2
Sleep apnea	4	4/84	4.7
Excessive alcohol consumption	1	1/84	1.2
Emphysema	1	1/84	1.2
Acute kidney failure	1	1/84	1.2
Weight gain	1	1/84	1.2
Chronic obstructive pulmonary disease	3	3/84	3.5
Gastro-esophageal reflux disease	2	2/84	2.3
Chronic cerebral vasculopathy	1	1/84	1.2
Anxiety-depressive	1	1/84	1.2
Schizophrenia	1	1/84	1.2
Acute kidney injury	1	1/84	1.2
Chronic kidney disease	1	1/84	1.2
Hemodialysis	1	1/84	1.2
Migraines	1	1/84	1.2
Hypothyroidism	1	1/84	1.2

n; number of patients with any variables; N; the total number of patients with CAD.

a ARDS; Acute respiratory distress syndrome.

**Table 5:** Sign and symptoms in COVID-19 patients with CAD based on case report/series studies.

Variable	No of study	n/N	%
Abdominal pain	3	3/84	3.5
Chest pain	15	19/84	22.3
Shortness of breath	9	11/84	12.9
Sore throat	2	2/84	2.3
Cough	25	29/84	34.1
Fever	32	40/84	47.0
Myalgia	11	11/84	12.9
Nausea	5	5/84	5.9
Diarrhea	6	7/84	8.2
Malaise	2	3/84	3.5
Chills	7	8/84	9.4
Dyspnea	21	21/84	24.7
Nocturia	1	1/84	1.2
Headache	4	4/84	4.7
Asthenia	2	2/84	2.3
Dizziness	1	1/84	1.2
Anosmia	2	2/84	2.3
Vomiting	4	4/84	4.7
Edema	3	3/84	3.5
Mottled legs	1	1/84	1.2
Angina	1	1/84	1.2
Weakness	1	1/84	1.2
Gastrointestinal symptoms	1	1/84	1.2
Dry mucous membranes	1	1/84	1.2
Flu like syndrome	2	2/84	2.3
Sweating	1	1/84	1.2
Hemiplegia	1	1/84	1.2
Aphasia	1	1/84	1.2
Anorexia	1	1/84	1.2
Tachycardia	3	3/84	3.5

n, number of patients with any variables; N, the total number of patients with CAD.

**Table 6:** Types of CAD of patients evaluated in included studies.

Variable	No of study	n/N	%
PCI^a^	3	3/84	3.5
STEMI^b^	5	5/84	5.9
ALCAPA^c^	1	1/84	1.2
CABG^d^	2	2/84	2.3
Coronary Atherosclerosis	3	6/84	7.0
Coronary Aneurysms	1	1/84	1.2
Occluded artery	5	5/84	5.9
Coronary artery dilatation	3	3/84	3.5
Severe multivessel coronary disease	3	3/84	3.5
Acute coronary syndrome	7	8/84	9.4
Left coronary artery apical aneurysm	1	1/84	1.2
Severe coronary artery stenosis	1	1/84	1.2
Acute thrombotic occlusion of the right coronary artery	6	6/84	7.0
stenosis of the proximal left-anterior descending coronary artery	6	6/84	7.0
Tight stenosis of the mid-portion of the left anterior descending artery	1	1/84	1.2
Chronic coronary syndrome	1	1/84	1.2
Wellens syndrom	1	1/84	1.2
Myocarditis	2	2/84	2.3
Myocardial dysfunction	1	3/84	3.5
Acute myocardial injury	1	1/84	1.2
Cardiac conduction system	2	2/84	2.3
Stress cardiomyopathy	1	3/84	3.5

n, number of patients with any variables; N, the total number of patients with CAD.

a PCI; Percutaneous Coronary Intervention.

b STEMI; ST-elevation myocardial infarction.

c ALCAPA; Anomalous left coronary artery from the pulmonary artery.

d CABG; Coronary artery bypass graft surgery.

**Table 7:** Laboratory findings reported from included case report/series studies.

Variable	No of study	n/N	%
Leukocytosis	3	3/84	3.5
Lymphopenia	3	4/84	4.7
Neutrophilia	1	1/84	1.2
High ketone levels	1	1/84	1.2
High CRP	12	12/84	14.1
High D-dimer	14	17/84	20
Increased troponin	9	13/84	15.3
Thrombocytopenia	1	1/84	1.2
Thrombocytosis	1	1/84	1.2
Elevated LDH	1	4/84	4.7
Elevated ESR	4	5/84	5.9
Elevated ferritin	9	10/84	11.7
Elevated SGPT	1	1/84	1.2
Low hemoglobin	1	1/84	1.2
Elevated inflammatory markers	3	3/84	3.5
Inflammatory syndrome	4	4/84	4.7
Elevated IL6	2	2/84	2.3
High cholesterol	2	2/84	2.3
High triglyceride	1	1/84	1.2
Elevated BNP^a^	2	3/84	3.5
Elevated AST	1	1/84	1.2
Elevated ALT	1	1/84	1.2
High cTnT^b^	3	3/84	3.5
Elevated creatine kinase	4	4/84	4.7
High creatinine	1	1/84	1.2
Elevated myoglobin	1	1/84	1.2
High LDL	1	1/84	1.2
High fibrinogen	3	3/84	3.5

n, number of patients with any variables; N, the total number of patients with CAD.

a BNP; Brain natriuretic peptide.

b cTnT; Cardiac troponin T.

**Table 8:** Treatment options used for patients based on evaluated case report/series studies.

Variable		No of study	n/N	%
**Antiviral**	Lopinavir/ritonavir	2	2/84	2.3
	Favipiravir	1	1/84	1.2
	Oseltamivir	2	2/84	2.3
	Remdesivir	2	2/84	2.3
	Lopinavir	2	2/84	2.3
	Ritonavir	2	2/84	2.3
	Darunavir	1	1/84	1.2
	Umifenovir	3	5/84	5.9
**Antibacterial**	Azithromycin	10	10/84	11.7
	Levofloxaicin	1	1/84	1.2
	Ceftriaxone	5	5/84	5.9
	Amoxicillin/Clavulanic acid	1	1/84	1.2
	Piperacillin-tazobactam	2	2/84	2.3
	Doxycycline	1	1/84	1.2
**Others**	Asprin	20	23/84	27.0
	Ticagrelor	3	3/84	3.5
	Hydrocortisone	1	1/84	1.2
	Hydroxychloroquine	14	16/84	18.8
	Tocilizumab	6	8/84	9.4
	Furosemide	4	5/84	5.9
	Methylprednisolone	5	5/84	5.9
	Clopidogrel	9	9/84	10.6
	Dexamethasone	3	3/84	3.5
	Enoxaparin	6	9/84	10.6
	Warfarin	1	1/84	1.2
	Heparin	1	1/84	1.2
	IVIG	6	6/84	7.0
	Corticosteroids	2	2/84	2.3
	Anakinra	2	2/84	2.3
	Tirofiban	2	2/84	2.3
	Apixaban	1	1/84	1.2
	Atorvastatin	2	2/84	2.3
	Metoprolol	4	4/84	4.7
	Prasugrel	2	2/84	2.3
	Colchicine	1	1/84	1.2
	Rituximab	1	1/84	1.2
	Cyclophosphamide	1	1/84	1.2
	Trazodone	1	1/84	1.2
	Fondaprinux	1	1/84	1.2
	Delorazepam	1	1/84	1.2
	Bisoprolol	1	1/84	1.2
	Ustekinumab	1	1/84	1.2
	Milirinone	1	2/84	2.3
	Nitroglycierin	2	2/84	2.3

n, number of patients with any variables; N, the total number of patients with CAD.

### 3.5 The prevalence of CAD-COVID-19 patients based on detection methods

Diagnosis of CAD-COVID-19 patients in most eligible studies was performed using real-time PCR, Echocardiography, Chest radiology, Angiography, and CT scan (Table 9). Based on the evaluated studies, Real-time PCR (48 studies) and echocardiography (17 studies) were the most common methods used to detect CAD-COVID-19 patients. The use of CT coronary angiogram (one study), electrocardiography, and CT angiography (Two studies for each) methods were also mentioned in fewer studies (Table 9).

**Table 9:** Diagnostic methods used in included studies.

Variable	No of study	n/N	%
PCR	46	72/84	84.7
Real time PCR	2	2/84	2.3
ELISA	4	4/84	4.7
CT Scan	7	9/84	10.6
Chest radiology	11	13/84	15.3
Echocardiography	17	24/84	28.2
Angiography	7	12/84	14.1
CT angiography	2	3/84	3.5
Electrocardiography	2	2/84	2.3
CT Coronary Angoigram	1	1/84	1.2

n, number of patients with any variables; N, the total number of patients with CAD.

## 4 Discussion

Increased risk of COVID-19 and subsequent exacerbation of the disease and even death from the virus appear to be associated with certain conditions, including heart disease, especially coronary artery problems. However, there is still little evidence on how coronary heart disease and COVID-19 relate. In various studies examining the role of cardiovascular disease during respiratory virus epidemics, people with cardiovascular disease have been associated with an increased risk of respiratory infections, including influenza. Patients were often at a greater risk of death [19]. In the present study, the first systematic review and meta-analysis about COVID-19 patients with coronary artery disease, prevalence studies, case reports, and case series were analyzed. Accordingly, a total of 20079 patients with COVID-19 were screened in prevalence studies; in addition, 84 patients in case reports and case series studies were also evaluated. Meta-analysis of prevalence studies showed that most COVID-19 patients were from continental Europe (mainly Italy, Norway, France, Serbia, Morocco, and Spain), and the prevalence of this disease was 12.4% (9.6–15.2). Also, the prevalence of this infection, according to studies published in Asia, was 11.7% (9.6–13.8), which was mainly reported in China and Oman. Studies conducted in the Americas found that the prevalence of COVID-19 was 10.8% (8.2–13.3), mainly in the United States. According to case reports and case series, the USA has the highest number of patients with simultaneous COVID-19 and CAD, with 35 reported cases. According to case reports and case series studies, it was found that the number of men was 2.52 times higher than women, and the average age of all patients was 52.3 years. These studies showed that the most common clinical symptoms in patients with COVID-19, who also had CAD, were fever, cough, dyspnea, and chest pain. On the other hand, cases such as decreased appetite, aphasia, hemiplegia, weakness, nocturia, and collapse were among the least reported symptoms in these patients. In a retrospective study conducted by Lian et al. [20] on 788 patients with COVID-19, the manifestations of the disease were examined in two groups: <60 years (n ​= ​652) and ≥60 years (n ​= ​136). It was noteworthy that most of the clinical symptoms of the infection were similar in both groups and included fever, cough, sputum, and fatigue. In the present study, evaluation of laboratory results of patients with CAD and COVID-19 revealed that there was a change in some important laboratory factors, including increased D-dimer, increased troponin, increased ferritin, and also an elevation in CRP levels. Evaluation of cardiac troponin I (cTnI) and cardiac troponin T (cTnT) necrotic biomarker is the gold standard for assessing myocardial damage worldwide. Elevated cardiac biomarkers are common in COVID-19 patients and it appears that an increase in cardiac troponin may be associated with disease severity and mortality from COVID-19 [21,22]. In a study by Guo et al. [23] on 187 hospitalized COVID-19 patients, cTnT levels were increased in 52 patients, all of whom had developed a myocardial injury. Mortality was higher in these patients compared to patients with normal cTnT levels (59.6% compared to 8.9%). Interestingly, a positive linear relationship was also observed in their study between cTnT and CRP, indicating an association between the severities of inflammation observed in COVID-19 and myocardial damage. The results of the study performed by Guan et al. [24] on 1099 COVID-19 patients showed an increase in D-dimer levels in 46% of all patients and, most importantly, in 60% of those with severe disease. According to a study by Shah et al. [25], out of 309 hospitalized COVID-19 patients, an increase in cTnI levels was observed in more than 1/3 of them (37.5%), most of whom had a mean age of 68.2 years and were male (53.5%). In an observational cohort study by Manocha et al. [26], the laboratory parameters of 446 out of 1053 COVID-19 patients in the USA were examined. Abnormal values and increases in TnI, CRP, Ferritin, and D-dimer were reported (50.7%, 97.8%, 90.6%, and 94%, respectively). Patients with severe TnI increase were elderly patients with significant coronary heart disease and prior stroke. Notably, in 112 patients with TnI level ≥0.34 ​ng/Ml, there was a significant increase in atrial tachyarrhythmias and ventricular tachyarrhythmias as well as mortality rate. The mean age of these patients was 69.7 years, and 70% were male. This study also revealed that a sharp increase in D-dimer and ferritin levels was associated with increased patient mortality. Evidence suggests that D-dimer levels rise during COVID-19, leading to coagulation disorders. D-dimer is a marker of fibrinolysis, and its increase is associated with venous thromboembolism and inflammation. The increase in this factor is associated with ICU hospitalization and increased risk of in-hospital mortality and acute myocardial injury [27]. These findings suggest that the severe form of SARS-CoV-2 infection is a systemic disease that involves multiple organs and subsequently leads to thrombosis and heart damage following inflammation. It can also be argued that changes in laboratory findings may be related to disease severity. However, the exact origin and cause of these laboratory changes in patients with COVID-19 cannot be ascertained. So far, despite countless efforts to control the COVID-19 pandemic, the disease remains a major challenge to the global community and a threat to public health, especially to susceptible individuals, including those with coronary artery disease. Although extensive research has been conducted worldwide to combat SARS-CoV-2, there is no definitive cure for the disease. However, supportive therapies such as oxygen therapy, control of fluid accumulation in the lungs, broad-spectrum antibiotics to prevent secondary bacterial infections, fluid intake to prevent dehydration, and medication for reducing fever may be helpful in the early stages of the disease. Umifenovir is a hemagglutinin inhibitor that can prevent fusion and virus entry into target cells. It can also inhibit viral RNA synthesis and regulate the immune system by inducing interferon production and activating phagocytes. A systematic review and meta-analysis by Huang et al. [28] on the efficacy and safety of Umifenovir in patients with COVID-19 showed that it had good tolerability and safety but limited efficacy. They stated that taking Umifenovir was associated with more reports of negative PCR on day 14 of COVID-19. In addition, they noted that Umifenovir could not significantly reduce hospital stays, improve symptoms, or reduce the risk of disease progression. The study conducted by Lian et al. had similar results [29]. However, according to a retrospective cohort study performed by Huang et al. [30] on COVID-19 patients, administration of chloroquine and Umifenovir reduced viral shedding intervals, reduced hospital stay, and consequently reduced hospital costs. They also found that taking Lopinavir/Ritonavir increased the viral shedding interval, increased hospital stay, and subsequently increased hospitalization costs and side effects. In a retrospective cohort study, Deng et al. [31] found that Umifenovir combined with Lopinavir/Ritonavir had a more favorable clinical response than Lopinavir/Ritonavir alone. Therefore, according to the results of most studies, there is no evidence to support the use of Umifenovir to improve disease outcomes in patients with COVID-19. The present study showed that in addition to antiviral drugs, other drugs such as azithromycin and HCQ were among the most widely used drugs in people with COVID-19. In addition, aspirin and clopidogrel were the most commonly used drugs in patients with CAD. Unfortunately, there is not enough information about the exact efficacy of most of the drugs listed in Table 8, so the efficacy of these drugs in the control and management of COVID-19 patients cannot be accurately determined. One of the most important aspects of the present study is the evaluation of underlying diseases in people with COVID-19 and coronary artery disease. According to the results of the present study, the most common underlying diseases in these individuals were hypertension, type 2 diabetes, and obesity (36.5%, 34.1%, and 12.9%, respectively). In a study conducted by Koutsoukis et al. [32] in France on 237 patients with myocardial infarction, some underlying diseases (mainly diabetes and hypertension) were more common in patients with myocardial infarction and COVID-19 than patients without COVID-19 (53.8% compared to 25.6%). In a systematic review and meta-analysis by Ssentongo et al. [33] on underlying diseases and their impact on COVID-19-induced death, the most common underlying diseases were cardiovascular disease, hypertension, high blood pressure, diabetes, and congestive heart failure. It has been shown that COVID-19 patients with underlying diseases have a higher mortality rate than those without underlying diseases. In the present study, the most common types of CAD were acute coronary syndrome, acute thrombotic occlusion of the right coronary artery, stenosis of the proximal left anterior descending coronary artery, occluded artery, and STEMI. There were also the fewest reports of ALCAPA, coronary aneurysms, Wellens syndrome, and stress cardiomyopathy. According to a review study by Helal et al. [34], a 28.1% decrease in the rate of hospitalization of patients with acute coronary syndrome during the COVID-19 pandemic was reported in 2020 compared to the same time in 2019 (61328 patients in 2020 compared to with 22539 patients in 2019). Also, a decrease of 21.9% in STEMI cases was reported in 2020 compared to 2019. Notably, this decrease was mainly observed in types of acute coronary syndrome with milder manifestations/symptoms (including NSTEMI and UA). This study also reported increased in-hospital cardiac arrest and frequent left ventricular systolic impairment. Another review study conducted by Roshdy et al. [35] on cardiac tissue autopsy in COVID-19-induced deaths found that cardiac abnormalities were common among these individuals. However, acute myocarditis changes were uncommon (1.5% of cases). The highest outcomes related to autopsy in COVID-19 patients included myocardial ischemia, thrombosis, and cardiac dilatation. These researchers also stated that SARS-CoV-2 was present in the hearts of almost half of the dead patients, but true myocarditis was observed in only 1.5% of the deceased patients. The results of the Kermani-Alghoraishi study [36] suggest that COVID-19, especially in severe forms, may play a role in the progression and development of acute coronary syndromes, especially STEMI, during increased thrombogenicity. Coronary artery angiography in these patients shows significantly large thrombosis and simultaneous involvement of several vessels. Given that in pandemics caused by Severe acute respiratory syndrome (SARS) and Middle East respiratory syndrome coronavirus (MERS-CoV), we have seen an increase in the rate of acute cardiovascular events in infected patients, hence such results in the SARS-CoV-2 pandemic was expected, but the results of many studies did not show such an event. Suggested factors that may explain this reduction in acute Coronary Syndrome during the COVID-19 pandemic include an increased commitment to medication, dietary changes, reduction in strain following quarantine in most communities, and increased people's cooperation in observing prevention and treatment protocols. Another important issue in the field of COVID-19 is the use of appropriate and accurate diagnostic methods. RT-PCR is the gold standard molecular diagnostic method for detecting this virus. This technique can be suitable for diagnosing infection in patients as well as screening infected carriers. However, it should be noted that this test is not very sensitive, so it will be very useful to combine several diagnostic methods to achieve sufficient sensitivity and specificity [37,38]. According to the present study's analysis, the most common diagnostic methods for identifying patients infected with COVID-19 were PCR, Chest radiology, CT scan, and ELISA. Additionally, based on published studies, echocardiography and angiography were the most frequently used methods to assess and control patients with CAD. Finally, it is important to note the limitations of the present study. First, only information about patients referred to the hospital was analyzed based on published articles. Second, some published articles failed to meet the inclusion criteria for our survey due to inadequate information (including clinical evidence, laboratory findings, and underlying diseases). Third, because there is no accurate and extensive information on COVID-19 patients with CAD in many parts of the world, we have not been able to fully document the exact prevalence of this group of patients worldwide. Fourth, in many studies, the outcome of COVID-19 in patients was not mentioned and therefore is not present in our study.

## 5 Conclusion

SARS-CoV-2 infection can be a burden for people with CAD. Due to the importance of assessing the status of people with CAD and COVID-19, various studies have been conducted in this field that has provided different information. The results of the present meta-analysis highlighted that in people with a history of CAD, especially men and elderly patients, it is important to note that if COVID-19-related symptoms occur and abnormal laboratory findings present, they should immediately refer to medical centers for on-time confirmation of the infection and time management to prevent the deterioration of their clinical condition. Investigation of the exact link between COVID-19 and CAD requires more extensive research. However, physicians should be aware of the potential effects of COVID-19 on patients with CAD and the consequences of viral infection.

## Funding

No funding.

## Ethical Approval

Not required.

## Credit author statement

**Bahareh Hajikhani**: designed the study, wrote and edited the manuscript, assumed overall responsibility for the accuracy and integrity of the manuscript, All authors contributed to the article and approved the submitted version, **Mahshid Safavi**: performed the data extraction, All authors contributed to the article and approved the submitted version, **Nazila Bostanshirin**: performed the data extraction, All authors contributed to the article and approved the submitted version, **Fatemeh Sameni**: performed the data extraction, All authors contributed to the article and approved the submitted version, **Mona Ghazi**: conducted the search strategy, All authors contributed to the article and approved the submitted version, **Shahrooz Yazdani**: performed the data extraction, did critical editing and revising of the manuscript, All authors contributed to the article and approved the submitted version, **Mohammad Javad Nasiri**: separately reviewed inclusion and exclusion criteria, All authors contributed to the article and approved the submitted version, **Nafiseh Khosravi-Dehaghi**: performed the data extraction, All authors contributed to the article and approved the submitted version, **Negin Noorisepehr**: separately reviewed inclusion and exclusion criteria, All authors contributed to the article and approved the submitted version, **Saba Sayyari**: performed the data extraction, All authors contributed to the article and approved the submitted version, **Masoud Dadashi**: designed the study, wrote and edited the manuscript, Formal analysis, did critical editing and revising of the manuscript, assumed overall responsibility for the accuracy and integrity of the manuscript, All authors contributed to the article and approved the submitted version.

## Declaration of competing interest

The authors declare no conflicts of interest.
